# Validation and Refinement of the Baveno VI Criteria for Ruling Out High-Risk Varices

**DOI:** 10.1155/2020/4217512

**Published:** 2020-11-27

**Authors:** Hong Zhou, Han Hu, Maoyong Tian, Jun Chu, Caiyun Tian, Yanqing Yang, Shide Lin

**Affiliations:** ^1^Department of Infectious Diseases, Affiliated Hospital of Zunyi Medical University, 201 Dalian Street, Guizhou 563003, China; ^2^Department of Infectious Diseases, Suining Central Hospital, 127 Desheng Street, Suining, Sichuan 629000, China; ^3^Department of Infectious Diseases, Xishui County People's Hospital, Xiangjiang Street, Xishui, Guizhou 564600, China

## Abstract

In the past decade, numerous studies have evaluated the roles of noninvasive methods for diagnosing or excluding varices and high-risk varices in patients with liver cirrhosis. The Baveno VI criteria recommend the use of a simple algorithm based on a liver stiffness measurement < 20 kPa through transient elastography and a platelet count > 150 × 10^9^/L for ruling out high-risk varices in patients with compensated advanced chronic liver disease. A large number of studies have validated the clinical usefulness of Baveno VI criteria for excluding high-risk varices. Several strategies have been proposed to refine the Baveno VI criteria; however, currently there is no review to summarize the diagnostic accuracy and limitations of the Baveno VI criteria after extensive validation. In this review, we summarize the diagnostic accuracy and limitations of the Baveno VI criteria after extensive validation. We also discuss methods to refine these criteria.

## 1. Introduction

Portal hypertension (PH) is defined by a hepatic venous pressure gradient (HVPG) > 5 mmHg [1]. PH is responsible for the development and progression of the majority of severe complications of liver cirrhosis, such as ascites, esophageal varices (EV), esophagogastric variceal bleeding (EVB), and hepatic encephalopathy [2]. EVB is a common complication in patients with liver cirrhosis associated with high mortality. Either beta-blocker therapy or band ligation for the primary prophylaxis of EVB has been confirmed to reduce the bleeding rate by 50% in patients with high-risk varices (HRV) (i.e., medium/large EV or the presence of red signs on varices of any grade) [3, 4]. Therefore, screening for HRV is mandatory in the management of patients with liver cirrhosis [2, 5, 6].

Screening through gastroscopy has been recommended for the assessment of EV and bleeding risk following the diagnosis of liver cirrhosis [2, 4, 7, 8]. However, gastroscopy is an invasive and expensive procedure associated with risks [9]. In the past decade, owing to advances in the noninvasive diagnostic methods of liver cirrhosis, an increased number of patients with liver cirrhosis were diagnosed at an early stage [10, 11]. The prevalence of HRV in patients with early liver cirrhosis is very low; hence, most screening gastroscopies yield negative findings. Noninvasive screening methods for the diagnosis or exclusion of EV or HRV are promising for the avoidance of unnecessary gastroscopies [12–14].

The Baveno VI criteria recommended using liver stiffness measurement (LSM) < 20 kPa through transient elastography (TE) and a platelet (PLT) count > 150 × 10^9^/L for ruling out HRVs in patients with compensated advanced chronic liver disease (cACLD) [7]. Following the introduction of the Baveno VI criteria, a large number of studies evaluated their value in excluding HRV in patients with cACLD or compensated liver cirrhosis.

In the evaluation of the usefulness of Baveno VI criteria for excluding HRVs, one of the most important index is the efficacy of Baveno VI criteria, which is reflected by the rate of spared endoscopy, that is, how many patients without HRVs will be spared from endoscopy; another important index is its safety, that is, how many patients with HRVs will be misclassified. Baveno VI criteria defined the acceptable threshold of the rate of missed HRV as 5%, but did not state the method used to calculate it. Three different methods to calculate missed HRV rate have been used [15]. In all of them, the numerator was the number of missed HRVs, but the denominator was either of the following: the number of spared endoscopies, the number of HRVs, or the whole patient population. The rate of missed HRVs/spared endoscopies corresponds to 1 − negative predictive value (NPV) of Baveno VI criteria and is the most used index; however, a recent study found that it did not correctly reflect the clinical benefit of Baveno VI criteria [15]. The rate of missed HRVs/total HRVs corresponds to 1 − sensitivity of Baveno VI criteria and provides the true proportion of missed HRVs. The rate of missed HRVs/the number of all patients provides the prevalence of missed HRVs. It is suggested that both the rates of missed HRVs/spared endoscopies and missed HRVs/total HRVs be reported in the evaluation of Baveno VI criteria [15]; however, most previous studies did not report the rate of missed HRVs/total HRVs. Therefore, in this review, we summarized both the rates of missed HRVs/spared endoscopies and missed HRVs/total HRVs to evaluate the benefits and the limitations of Baveno VI criteria for excluding HRVs.

## 2. Pathogenesis and Epidemiology of PH and Varices

The prevalence of EV among patients with compensated cirrhosis is about 30%~40%, whereas it is up to 85% among patients with decompensated cirrhosis. In patients with compensated cirrhosis, EV develops at a rate of 7%~8% per year, and progresses from small to large varices at a rate of 10%~12% per year. The incidence of EVB is approximately 10% to 15% per year in patients with HRV. Six-week mortality of EVB ranges between 15% and 25% [2].

In patients with liver cirrhosis, the portal pressure is increased due to the increased intrahepatic resistance and portal system blood flow [16]. The structural alterations in the cirrhotic liver, such as sinusoidal fibrosis, regenerative nodules, and functional vasoconstriction of the intrahepatic circulation, contribute to the increase in intrahepatic resistance [17]. Increased intrahepatic fibrosis is the primary factor leading to increased HVPG in the early stage of progression of liver fibrosis. At this stage, there is a good correlation between the grades of liver fibrosis and levels of portal pressure [18]. The HVPG critical threshold of 10 mmHg has been defined as clinically significant PH owing to the occurrence of complications of PH over this threshold. When the HVPG reaches this threshold, the increased portal system blood flow (as a result of splanchnic arterial vasodilation) further aggravates PH [19]. Therefore, when HVPG exceeds 10 mmHg, the level of portal pressure is not completely correlated with the grades of liver fibrosis [20].

Anatomical, physical, and biological factors play an important role in the development and progression of esophageal and gastric varices [17, 21]. Varices are considered the result of the opening and dilatation of vessels between the portal and systemic circulation due to the increased portal pressure [20]. Recent studies suggested that active angiogenesis also modulated the formation of varices [22, 23]. The risks of EVB are determined by the pressure of the intravariceal and esophageal lumen, as well as the radius and the thickness of the variceal wall. As the portal pressure increases, the variceal size increases and the wall thickness decreases. Therefore, the most important determining factor of EVB is the level of HVPG [24, 25]. HVPG > 12 mmHg is a strong predictor of EVB. As the Child-Pugh C class often reflects a higher portal pressure and red color signs on gastroscopy typically indicate an area with thin variceal wall, they are also risk factors for EVB [23].

## 3. The Validation of Baveno VI Criteria

In patients with chronic liver diseases, the progression of liver fibrosis is a continuous process. cACLD is a significant stage in this process, as clinically significant PH and varices may develop during this stage [26, 27]. The Baveno VI consensus recommended using LSM through TE to identify cACLD. LSM = 10–15 kPa andLSM > 15 kPa are suggestive (needing further test for confirmation) and highly suggestive of cACLD, respectively [7].

Screening for HRV is important for evaluating the prognosis and selecting appropriate treatment methods for patients with cACLD [28]. The Baveno VI criteria recommended using LSM < 20 kPa through TE and a PLT count > 150 × 10^9^/L for ruling out HRVs in patients with cACLD [7]. Several advantages associated with the use of the Baveno VI criteria to stratify the risk of EV have been reported.

One of the major advantages of using the Baveno VI criteria to rule out HRV is their high reliability and safety. As shown in Table 1, applying this standard to patients with cACLD or compensated liver cirrhosis resulted in a risk to miss HRV < 5%, despite the rate of spared gastroscopies being approximately 8.1–46.2%. In a recent meta-analysis, which included 30 studies (8469 participants), the Baveno VI criteria for excluding HRVs had a pooled sensitivity and specificity of 97% and 32%, respectively. Among 1000 patients with cACLD and a 20% prevalence of HRVs, the Baveno VI criteria would avoid 262 gastroscopies and miss only six patients with HRVs. Another meta-analysis, including 13 studies and 4464 patients with cACLD, yielded similar results; the pooled rate of missing HRV was 0.3% and 32.8% of the gastroscopies could be avoided. The sensitivity, specificity, and area under the receiver operating curve of the Baveno VI criteria were 97%, 41%, and 96%, respectively [29]. These studies demonstrated that the Baveno VI criteria is a reliable method for stratifying the risk of EV and sparing gastroscopies in patients with cACLD or compensated cirrhosis.

Another advantage of the Baveno VI criteria is that they maintain high discriminating accuracy for ruling out HRVs in patients with different etiologies. The Baveno VI criteria for diagnosing cACLD or ruling out HRV have been based on data obtained mostly from patients with hepatitis C virus (HCV) or alcohol etiology. A few studies found that the optimal thresholds of LSM for diagnosing liver cirrhosis and discriminating EV and HRV are different between patients with different etiologies [30, 31]. However, subsequent studies reported that the Baveno VI criteria could be used to safely rule out HRVs in patients with nonalcoholic fatty liver disease (NAFLD), primary biliary cholangitis (PBC), and primary sclerosing cholangitis (PSC). Petta et al. evaluated the Baveno VI criteria in patients with NAFLD-related compensated cirrhosis [32]. The results showed that gastroscopies could be spared in 106 of the 314 patients (33.8%) and only one patient (0.9%) had HRV. Moctezuma-Velazquez et al. retrospectively investigated 227 patients with cACLD due to PBC and PSC, with a 13% prevalence of HRV. The Baveno VI criteria spared 39% and 30% of gastroscopies in patients with PBC and PSC, respectively, without missing HRV [33]. In a recent meta-analysis involving patients with cACLD due to HCV, HBV infection, NAFLD, and alcohol liver disease, the Baveno VI criteria missed HRVs at a rate of 0.0%, 1.2%, 0.0%, and 0.0%, respectively [29]. In addition, gastroscopies were spared in 24.2%, 24.9%, 38.6%, and 27.0% of patients, respectively. Another meta-analysis including 30 studies and 8469 participants also found similar results [34]. In HBV-, HCV-, alcohol-, and NAFLD-related cACLD, the pooled sensitivity and specificity of the Baveno VI criteria for HRVs ranged 93–99% and 30–37%, respectively.

As demonstrated by two recent preliminary studies, the Baveno VI criteria can be used for the screening and surveillance of PH in patients with HBV- or HCV-associated liver cirrhosis receiving antiviral therapy. In patients with HCV- or HBV-related cirrhosis, elimination of HCV and inhibition of HBV DNA replication through antiviral treatment are associated with the improvement of fibrosis and a decline in portal pressure, finally resulting in a significant decrease in liver-related complications [35, 36]. The Baveno VI criteria were established based on patients with active HCV or HBV replication. The predictive value for HRVs in patients with sustained virological response (SVR) to antiviral therapy remains unknown. Thabut et al. evaluated the Baveno VI criteria in patients with compensated liver cirrhosis due to HBV or HCV infection, and with or without a sustained response to antiviral therapy. They found that, at the time of PH progression (as the onset of HRV- or PH-related bleeding), all patients exhibited worsening of their Baveno VI status [37]. In another study, Puigvehi et al. investigated 230 patients with liver cirrhosis due to HCV and SVR after treatment with direct-acting antivirals. The NPV of the Baveno VI criteria to exclude HRV was maintained after SVR [38]. These results suggested that the Baveno VI criteria can be used for the screening and surveillance of PH in patients with HBV- or HCV-associated liver cirrhosis receiving antiviral therapy.

## 4. Limitation of the Baveno VI Criteria

As shown in Table 1, the Baveno VI criteria spare only approximately 8.1–46.2% of the gastroscopies. Of note, >40% of unnecessary gastroscopies cannot be spared in patients with cACLD. This is attributed to the low specificity of the Baveno VI criteria for ruling out HRV, which results in a large number of unnecessary gastroscopies. In a meta-analysis, 74% of patients with cACLD did not meet the Baveno VI criteria and were referred for screening endoscopy to detect the possible presence of HRVs. However, the majority of these patients (74%) did not have HRVs [34].

Several factors that may influence the rate of spared gastroscopies have been investigated. A meta-analysis found a negative correlation of the proportion of viral liver disease, as well as the levels of alanine amino transferases (ALT) and aspartate amino transferases, with the rate of spared endoscopy [29]. In patients with HBV infection, the diagnostic thresholds of LSM for cACLD and liver cirrhosis are lower than those in patients with other etiologies. Therefore, the risk of having HRVs in HBV patients with LSM < 20 kPa and a PLT count > 150 × 10^9^/L is higher than that in patients with other etiologies [39]. A positive correlation between the proportion of NAFLD and rate of spared endoscopy was reported [29]. In a previous study, we found that, among patients with HBV-related compensated liver cirrhosis who did not meet the Baveno VI criteria, the prevalence of HRV was significantly lower in those with ALT or total bilirubin (TBil) ≥ 2 upper limit of normal (ULN) (14.3%) than in those with both ALT and TBil < 2 ULN (34.1%) [40]. These findings suggested that the concomitant liver inflammation may falsely increase LSM, making it difficult for patients to fulfill the Baveno VI criteria and decreasing the rate of spared gastroscopies.

Another limitation of the Baveno VI criteria is that LSM may not be reliable in ≤20% of patients. LSM is affected by the position of the probe [41], concomitant liver inflammation [42], intra- and extrahepatic cholestasis [43], body mass index [44], the experience of the operator, etc. [45, 46]. In addition, LSM also showed high variation, especially in patients with high LSM values [47]. A recent study suggested that performing two LSMs on different days, as recommended by the Baveno VI criteria, may improve the diagnostic accuracy [47]. However, undergoing two TE examinations within a short period of time may not be acceptable to patients. Among a large number of studies concerned with the evaluation of the Baveno VI criteria, few studies had implemented the two-LSM approach.

Several factors, other than PH, may also have a significant impact on PLT. In a study reported by Maurice et al., two patients with HRV were missed by the Baveno VI criteria, and one of those had a previous splenectomy [48]. Furthermore, LSM through TE may not be easily available in developing countries, limiting the use of the Baveno VI criteria.

As shown in Table 1, the other limitation of the Baveno VI criteria is that in a few studies the rate of missed HRVs/total HRVs exceeded 5% [48–51]. If more stringent criteria of the missed HRVs/total HRV < 5% is adopted, the Baveno VI criteria is unreliable in these studies. The rate of missed HRVs/total HRVs corresponds to sensitivity (1 − sensitivity) for HRVs and provides the true proportion of missed HRVs. These findings indicate that the sensitivity of Baveno VI criteria is not as high as its NPV for excluding HRVs.

## 5. Refinement of the Baveno VI Criteria

Several studies have attempted to overcome the limitations of the Baveno VI criteria. The strategies applied for the refinement of these criteria include adjustment of the LSM and PLT thresholds and combination with other predictors (Table 2).

Several thresholds of the LSM (25–30 kPa) and PLT count (100–120 × 10^9^/L) have been examined to increase the rate of spared gastroscopies. The most broadly studied criteria were LSM 25/PLT 110 (Expanded Baveno VI). Augustin et al. increased the threshold of LSM to <25 kPa and decreased that of the PLT count to >110 × 10^9^/L in patients with cACLD of different etiologies [52]. The Expanded Baveno VI criteria increased the rate of spared gastroscopies from 21% to 40% compared with the Baveno VI criteria, with a 0.6% risk of missing HRV. The refined criteria also performed well in patients with cACLD of different etiologies. However, the rate of missed HRVs/total HRVs increased to 6.5%. Bae et al. found that, in patients with cACLD due to HBV infection, the Expanded Baveno VI criteria could spare more gastroscopies than the Baveno VI criteria (51.7% vs. 27.6%, respectively) and could miss more HRVs (6.8% vs. 3.8%, respectively) [51]. In another study including patients with chronic HBV infection, the Expanded Baveno VI criteria missed >5% of HRVs [53]. In a meta-analysis, Stafylidou et al. found that the Expanded Baveno VI criteria could further reduce the rate of unnecessary gastroscopies compared with the Baveno VI criteria (42.8% vs. 26.2%, respectively) and could be associated with a higher rate of missed HRVs (5% vs. 2%, respectively) [34].

Ding et al. found that gastroscopies could be avoided in a total of 107 patients (39%) with the combination of LSM ≤ 25 kPa and a PLT count ≥ 100 × 10^9^/L, having a NPV of 100% [54]. However, in another study of patients with HCV infection, these criteria missed 10% of the total HRVs [55].

Several studies attempted to identify the optimal excluding criteria for HRVs in patients with different etiologies. Petta et al. proposed a set of NAFLD cirrhosis criteria: PLT count > 110 × 10^9^/L and LSM < 30 kPa for the M probe, and PLT count > 110 × 10^9^/L and LSM < 25 kPa for the XL probe [32]. Use of these criteria led to an absolute reduction in the number of gastroscopies by 34.7% and 10.5% compared with the Baveno VI and Expanded Baveno VI criteria, respectively; however, the rate of missed HRVs/total HRVs reached as high as 31.3%. Lee et al. found that the optimal criteria for ruling out HRVs in patients with chronic HBV infection were LSM < 20 kPa and PLT count > 120 × 10^9^ cells/L [53]. These criteria spared 36.2% of gastroscopies and missed only 4.6% of HRVs. In patients with alcoholic liver disease, LSM < 20 kPa and PLT count > 110 × 10^9^/L or LSM < 25 kPa and PLT count > 120 × 10^9^ cells/L spared 30.1% and 29.5% of gastroscopies and missed 4.7% and 1.9% of HRVs, respectively.

In our previous study [40], we found that ALT was independently negatively associated with the prevalence of HRV in patients with HBV-related compensated cirrhosis who did not fulfill the Baveno VI criteria. In patients with ALT or TBil ≥ 2 ULN, the Lok index and PLT yielded an area under the receiver operating curve of 0.814 and 0.741, respectively. Lok index ≤ 0.5596 or PLT count > 100 × 10^9^/L further spared 39.6% and 43.9% of gastroscopies, respectively, without missing HRVs. In the patients with ALT and TBil < 2 ULN, LSM < 20.6 kPa further spared 39.0% of gastroscopies without missing HRVs. The results of our study suggested that LSM, PLT, or the Lok index (stratified according to ALT and TBil) accurately identified more patients without HRV. However, as the number of patients included in this study was small, the results also require validation in a study with a larger sample size.

Spleen stiffness measurement (SSM) through TE or magnetic resonance elastography (MRE), as well as the combination of SSM, PLT count, spleen size, and LSM, has also been evaluated for the identification of patients with HRV [56–59]. A few studies have reported the superior diagnostic accuracy of SSM versus LSM and PLT for the prediction of EV and HRV [60–62]. Colecchia et al. found that the combination of SSM ≤ 46 kPa with the Baveno VI criteria avoided 43.8% of gastroscopies (Baveno VI criteria: 21.7%), with <5% of HRVs missed [63]. These results were confirmed in a prospective external validation cohort, as the combined Baveno VI/SSM ≤ 46 model safely spared 37.4% of gastroscopies (0 HRV missed) compared with 16.5% when using the Baveno VI criteria alone.

As the spleen is often stiffer than the liver, spleen stiffness cannot be measured using TE with an upper limit of 75 kPa in some patients. Stefanescu et al. used SSM@100 Hz (with the upper limit of 100 kPa) instead of SSM@50 Hz (assessed by the standard liver-dedicated TE, with a probe of 50 Hz) as a new noninvasive marker for EV and HRV to overcome this limitation [64]. They found that SSM@100 Hz provided a higher accuracy than other noninvasive methods. The sequential combination of SSM@100 Hz with the Baveno VI criteria spared 38.9% of unnecessary gastroscopies (Baveno VI criteria alone: 8.1%), and the missed HRV rate was 4.7%.

Matsui et al. found that LSM through MRE significantly decreased the unsuccessful rate (0.3%) compared with that observed for TE (19.6%) in certain patients, such as those with a high body mass index or with ascites [65]. However, LSM through MRE was not associated with an increase in the rate of spared gastroscopies compared with the Baveno VI criteria.

Several studies attempted to predict HRV without using LSM or SSM to overcome the limitation of unavailable TE in some hospitals. Jangouk et al. recently reported a 12% increase in the rate of spared gastroscopies (without additional HRV missed) by expanding the Baveno VI criteria to patients with model for end − stage liver disease (MELD) = 6 [66]. In addition, a stepwise strategy using PLT count > 150 × 10^9^ cells/L and MELD = 6 without LSM substantially increased the number of avoided gastroscopies, maintaining a very low rate of missed HRVs. Although some studies confirmed the accuracy of this approach [67], a recent study reported contradictory results (missing approximately 10% of HRVs) [68]. Calvaruso et al. found that, among 1381 patients with HCV-associated cirrhosis, a PLT count cut-off value of >120 × 10^9^/L and a serum albumin level > 36 g/L were able to identify patients without medium/large EV [69]. Moreover, the NPV was slightly higher than that observed for the Baveno VI and Expanded Baveno VI criteria.

Calès et al. developed a strategy for the diagnosis of large esophageal varices in patients with compensated liver cirrhosis [70], which involved the sequential combination of a blood test and esophageal capsule endoscopy (ECE). This strategy significantly increased spared endoscopy rates compared to that of the Baveno VI criteria. However, its applicability requires validation and ECE cost optimization.

Recently, Protopapas et al. used PLT/log_10_LSM to refine Baveno VI criteria [71], and they found that PLT/log_10_LSM ≤ 122,000 *μ*L^−1^ × kPa^−1^ predicted HRV with 100% sensitivity and negative predictive value (NPV), sparing 22 (20.6%) of the patients from unneeded screening endoscopy without missing HRVs.

As shown in Table 2, although most of these refining criteria improved the rate of spared gastroscopies, they also missed more HRVs than that of Baveno VI criteria, especially the rate of missed HRVs/total HRVs which showed a great variability in these studies. In addition, most of these refining criteria still need extensive validation in future studies.

## 6. Conclusion

After extensive validation, the Baveno VI criteria exhibited high reliability for safely ruling out HRVs and avoiding unnecessary gastroscopies. However, its efficacy for excluding patients with HRVs remains unsatisfactory: >40% of unnecessary gastroscopies cannot be spared through the use of the Baveno VI criteria in patients with cACLD. Several strategies have been proposed to refine the Baveno VI criteria, and most of them improved the rate of spared gastroscopies. However, their accuracy requires further validation.

In clinical practice, Baveno VI criteria is currently the only reliable method and can be used to screen HRVs in patients with compensated liver cirrhosis with most common etiologies. The refined Baveno VI criteria or the criteria without LSM is not suitable for screening HRVs.

## Figures and Tables

**Table 1:** Validation of the Baveno VI criteria.

Author	Research population (*n*, etiology)	Prevalence of HRV (*n*, %)	Spared endoscopy	Missed HRVs/saved endoscopies	Missed HRVs/total HRVs
Maurice [48]	310, mixed	15 (5%)	102/310 (33.0%)	2/102 (2.0%)	2/15 (13.3%)
Jangouk [66]	262, mixed US cohort (161)	14 (9%)	41/161 (25.4%)	0/41 (0%)	0/14 (0%)
	Italian cohort (101)	17 (17%)	16/101 (15.8%)	0/16 (0%)	0/17 (0%)
Sousa [72]	104, mixed	9 (9%)	48/104 (46.2%)	0/48 (0%)	0/9 (0%)
Wong [58]	127, mixed	11 (9%)	105/127 (82.7%)	1/105 (1.0%)	1/11 (9.1%)
Petta [32]	790, NAFLD				
	Training cohort (314)	32 (10%)	106/314 (33.8%)	1/106 (0.9%)	1/32 (3.1%)
	Validation cohort (338)	45 (13%)	113/338 (33.4%)	5/113 (4.4%)	5/45 (11.1%)
Bae [51]	282, mixed	55 (20%)	78/282 (27.6%)	3/78 (3.8%)	3/55 (5.5%)
Colecchia [63]	498, mixed				
	Internal cohort (240)	46 (19%)	52/240 (21.7%)	1/52 (1.9%)	1/46 (2.2%)
	External cohort (115)	15 (13%)	19/115 (16.5%)	0/19 (0%)	0/15 (0%)
Tosetti [68]	442, mixed	31 (7%)	86/442 (19.5%)	0/86 (0%)	0/31 (0%)
Lee [53]	1218, mixed	249 (20%)	313/1218 (25.7%)	6/313 (1.9%)	6/249 (2.4%)
Stefanescu [64]	185, mixed	43 (23%)	15/185 (8.1%)	0/15 (0%)	0/43 (0%)
Gaete [73]	300, mixed	54 (18%)	95/300 (31.7%)	1/95 (1.1%)	1/54 (1.9%)
Moctezuma-Velazquez [33]	227, PBC, PSC	30 (13%)	82/227 (36.1%)	0/82 (0%)	0/30 (0%)
Protopapas [71]	107, mixed	22 (21%)	13/107 (12.1%)	0/13 (0%)	0/22 (0%)

Note: HRV—high-risk varices; NAFLD—nonalcoholic fatty liver disease; PBC—primary biliary cholangitis; PSC—primary sclerosing cholangitis.

**Table 2:** Refinement of the Baveno VI criteria.

Author	Patients	Criteria	Prevalence of HRV (*n*, %)	Spared endoscopy (%)	Missed HRVs/saved endoscopies	Missed HRVs/total HRVs
Ding [54]	271, mixed	LSM ≤ 25 + PLT ≥ 100	26 (10%)	107/271 (39%)	0/107 (0%)	0/26 (0%)
Puigvehi [55]	368, HCV	LSM ≤ 25 + PLT ≥ 100	55 (15%)	149/368 (40.5%)	10/149 (6.7%)	10/55 (18.2%)
Jangouk [66]	262, mixed					
	US cohort (161)	Baveno VI	14 (9%)	41/161 (25.4%)	0/41 (0%)	0/14 (0%)
		Baveno VI + MELD = 6	14 (9%)	60/161 (37.3%)	0/60 (0%)	0/14 (0%)
		PLT > 150 + MELD = 6	14 (9%)	86/161 (53.4%)	0/86 (0%)	0/14 (0%)
	Italian cohort (101)	Baveno VI	17 (17%)	16/101 (15.8%)	0/16 (0%)	0/17 (0%)
		Baveno VI + MELD = 6	17 (17%)	28/101 (27.7%)	0/28 (0%)	0/17 (0%)
		PLT > 150 + MELD = 6	17 (17%)	30/101 (29.7%)	1/30 (3.3%)	1/17 (5.8%)
Augustin [52]	925, mixed	LSM < 25 + PLT > 110	92 (10%)	367/925 (40%)	6/367 (1.6%)	6/92 (6.5%)
		LSM < 25 + PLT > 110 + MELD = 6	92 (10%)	405/883 (45.8%)	7/405 (1.7%)	7/92 (7.6%)
Petta [32]	790, NAFLD	(M probe) training cohort:				
		Baveno VI	32 (10%)	106/314 (33.8%)	1/106 (0.9%)	1/32 (3.1%)
		LSM < 25 + PLT > 110	32 (10%)	182/314 (58%)	7/182 (3.8%)	7/32 (21.9%)
		LSM < 30 + PLT > 110	32 (10%)	215/314 (68.5%)	9/215 (4.2%)	9/32 (28.1%)
		Validation cohort:				
		Baveno VI	45 (13%)	113/338 (33.4%)	5/113 (4.4%)	5/45 (11.1%)
		LSM < 25 + PLT > 110	45 (13%)	183/338 (54.1%)	8/183 (4.4%)	8/45 (17.8%)
		LSM < 30 + PLT > 110	45 (13%)	209/338 (61.8%)	10/209 (4.8%)	10/45 (22.2%)
		(XL probe) training cohort:				
		LSM < 25 + PLT > 110	32 (10%)	204/314 (65%)	10/204 (4.9%)	10/32 (31.3%)
		Validation cohort:				
		LSM < 25 + PLT > 110	14 (10%)	64/138 (46.4%)	1/64 (1.6%)	1/14 (7.1%)
Bae [51]	282, mixed	Baveno VI	55 (20%)	78/282 (27.6%)	3/78 (3.8%)	3/55 (5.5%)
		LSM < 25 + PLT > 110	55 (20%)	146/282 (51.7%)	10/146 (6.8%)	10/55 (18.2%)
Colecchia [63]	498, mixed	Internal cohort:				
		Baveno VI	46 (19%)	52/240 (21.7%)	1/52 (1.9%)	1/46 (2.2%)
		SSM ≤ 46	46 (19%)	86/240 (35.8%)	1/86 (1.2%)	1/46 (2.2%)
		Baveno VI + SSM ≤ 46	46 (19%)	105/240 (43.8%)	2/105 (1.9%)	2/46 (4.3%)
		External cohort:				
		Baveno VI	15 (13%)	19/115 (16.5%)	0/19 (0%)	0/15 (0%)
		SSM ≤ 46	15 (13%)	35/115 (30.4%)	0/35 (0%)	0/15 (0%)
		Baveno VI + SSM ≤ 46	15 (13%)	43/115 (37.4%)	0/43 (0%)	0/15 (0%)
Tosetti [68]	442, mixed	Baveno VI	31 (7%)	86/442 (19.5%)	0/86 (0%)	0/31 (0%)
		LSM < 25 + PLT > 110	31 (7%)	193/442 (43.7%)	0/193 (0%)	0/31 (0%)
		LSM < 25 + PLT > 125	31 (7%)	154/442 (34.8%)	0/154 (0%)	0/31 (0%)
		PLT > 150 + MELD = 6	31 (7%)	171/442 (38.7%)	3/171 (1.8%)	3/31 (9.7%)
Lee [53]	1218, mixed	Baveno VI	249 (20%)	313/1218 (25.7%)	6/313 (1.9%)	6/249 (2.4%)
		LSM < 20 + PLT > 110	249 (20%)	476/1218 (39.1%)	21/476 (4.4%)	21/249 (8.4%)
		LSM < 25 + PLT > 120	249 (20%)	476/1218 (39.1%)	23/476 (4.8%)	23/249 (9.2%)
		LSM < 25 + PLT > 110	249 (20%)	530/1218 (43.5%)	31/530 (5.8%)	31/249 (12.4%)
		LSM < 30 + PLT > 150	249 (20%)	381/1218 (31.3%)	16/381 (4.2%)	16/249 (6.4%)
		LSPS < 1.47	249 (20%)	536/1218 (44.0%)	23/536 (4.3%)	23/249 (9.2%)
Stefanescu [64]	185, mixed	Baveno VI	43 (23%)	15/185 (8.1%)	0/15 (0%)	0/43 (0%)
		Baveno VI + SSM@50 Hz : 40.1	43 (23%)	49/185 (26.5%)	2/49 (4.1%)	2/43 (4.7%)
		Baveno VI + SSM@100 Hz : 41.3	43 (23%)	72/185 (38.9%)	2/72 (2.8%)	2/43 (4.7%)
Protopapas [71]	107, mixed	PLT/log_10_LSM	22 (21%)	22/107 (20.6%)	0/22 (0%)	0/22 (0%)

Note: HCV—hepatitis C virus; HRV—high-risk varices; LSM—liver stiffness measurement; LSPS—liver stiffness-spleen diameter to platelet ratio score; MELD—model for end-stage liver disease; NAFLD—nonalcoholic fatty liver disease; PLT—platelet; SSM—spleen stiffness measurement.

## Data Availability

The data used to support the findings of this study are included within the article.
